# GLP-1 receptor agonists in stroke prevention: a narrative review on emerging therapeutic frontiers

**DOI:** 10.1080/07853890.2026.2660386

**Published:** 2026-04-18

**Authors:** Rahul Chikatimalla, Ashka Shah, Twinkle Shah, Griffin Perry, Himanshi Banker, Kanishk Aggarwal, Rohit Jain

**Affiliations:** a Kamineni Institute of Medical Sciences, Narketpally, India; b GMERS Medical College, Gotri, Vadodara, India; c GMERS Medical College, Valsad, India; d Department of Medicine, Penn State Health Milton S. Hershey Medical Center, Hershey, PA, USA; e Maulana Azad Medical College, New Delhi, India; f Dayanand Medical College and Hospital, Ludhiana, Punjab, India; g Division of Hospital Medicine, Penn State Health Milton S. Hershey Medical Center, Hershey, PA, USA

**Keywords:** Stroke, type 2 diabetes mellitus, glucagon-like peptide 1 receptor agonists, cerebrovascular circulation, neuroprotection

## Abstract

**Objectives:**

To evaluate the current evidence supporting the cerebrovascular protective effects of glucagon-like peptide-1 receptor agonists (GLP-1RAs) in individuals with type 2 diabetes mellitus (T2DM), and to outline their mechanisms of action in stroke prevention.

**Methods:**

A narrative review was conducted by synthesising data from cardiovascular outcome trials, meta-analyses and mechanistic studies involving GLP-1RAs such as semaglutide, liraglutide and dulaglutide. The search included literature on ischaemic stroke incidence, molecular pathways and clinical outcomes associated with GLP-1RA therapy.

**Results:**

GLP-1RAs exhibit multiple protective mechanisms, including anti-inflammatory, antioxidant, neuroprotective and endothelial-stabilising effects. Long-acting agents demonstrate superior efficacy in reducing nonfatal and ischaemic stroke risk, with relative risk reductions ranging from 15% to 39% across major trials. These benefits are observed independent of glycemic control and appear most prominent in patients with preserved renal function and shorter diabetes duration. In contrast, short-acting exendin-based GLP-1RAs show limited cerebrovascular benefit. Treatment response may vary based on factors such as stroke subtype, baseline vascular risk and comorbidities.

**Conclusion:**

GLP-1RAs offer significant promise as adjunctive pharmacotherapy for stroke prevention in individuals with T2DM. Their multifactorial benefits extend beyond glucose regulation and may influence clinical outcomes through systemic vascular and neuroprotective mechanisms. However, inconsistencies in trial outcomes and limited data in non-diabetic or high-risk populations underscore the need for targeted stroke-specific studies. Personalised treatment approaches and broader risk stratification may optimise their use in cerebrovascular disease management.

## Key messages

GLP-1 receptor agonist (GLP-1RA) therapy should be incorporated into a broad approach for risk reduction for stroke in patients with type 2 DM, especially in situations where prevention of ischaemic stroke is of high importance.

Long-acting GLP-1 receptor agonists (e.g., semaglutide and dulaglutide) are preferred over shorter-acting preparations for their cerebrovascular protective effects, properties of which are more consistent and beneficial for the risk of ischaemia.

GLP-1RA therapy could provide a special advantage to patients with multiple risk factors for cardiometabolic diseases such as obesity, hypertension, dyslipidemia and documented atherosclerotic cardiovascular disease.

On the other hand, the neuroprotective properties of GLP-1RAs, which occur through anti-inflammatory, antioxidant, endothelial-stabilising or mitochondrial-protective actions, provide rationale for the use.

Treatment should be individualised for renal function, tolerance, potential for compliance, cost and accessibility. This allows for maximal long-term cerebrovascular benefits.

## Introduction

A stroke is a cerebrovascular incident marked by an interruption in blood supply to the brain, resulting in neurological impairments. Strokes are classified into two types: ischaemic strokes, which account for 87% of all cases and are caused by thrombotic or embolic blockages in cerebral arteries, and haemorrhagic strokes, which account for 13% and occur when blood vessels rupture, as seen in intracerebral and subarachnoid haemorrhages [1]. According to 2021 data, stroke impacted 11.9 million individuals and had a prevalence of 93.8 million, ranking as the third largest cause of death and the fourth leading contributor to disability-adjusted life years (DALYs) lost. The bulk of these occurrences occurred in low- and middle-income countries (LMIC) [2]. Between 2020 and 2022, stroke prevalence in the United States was 7.7% among people aged ≥65, 3.8% among those aged 45–64, and 0.9% among those aged 18–44. The overall prevalence was equal among males and females, with both reporting a rate of 2.9% [3]. The global age-standardized incidence rate of ischaemic stroke is expected to rise to 89.32 per 100,000 people by 2030, therefore highlighting the ongoing increase in its worldwide burden [4].

Stroke-related expenses are responsible for approximately 34% of global healthcare spending [5]. Each stroke survivor carries an adjusted annual direct cost of $4,317 (95% CI: $3,828–$4,807). Based on 2016 US dollar values, estimated indirect costs of underemployment and premature death were $38.1 billion and $30.4 billion, respectively, representing nearly 66% of the total economic burden of $103.5 billion [6]. The rising financial cost of stroke in developing countries may be affected by factors such as race, ethnicity, population size and varying differentials in access and affordability of healthcare services [7,8]. This economic burden is expected to increase since the majority of low- and middle-income countries (LMICs) are shifting from infectious to noncommunicable diseases as leading causes of disease burden [9].

Risk for stroke is influenced by both modifiable, e.g. smoking, diabetes mellitus, hypertension, obesity, inactivity, lack of exercise and atrial fibrillation – and non-modifiable factors, including genetics, age, sex and race/ethnicity (Boehme AK, 2017). One of the key modifiable risk factors for stroke is diabetes mellitus (DM). There are two main types of diabetes mellitus (DM): type 1 and type 2, each with distinct pathophysiology, clinical presentation and management strategies. Type 1 diabetes mellitus (T1DM) (HR: 1.50; 95% CI: 1.23–1.83) and type 2 diabetes mellitus (T2DM) (HR: 1.76; 95% CI: 1.65–1.87) both increase the risk of stroke compared to non-diabetic population [10]. T2DM significantly contributes to accelerated atherogenesis, endothelial dysfunction and thrombosis due to chronic hyperglycaemia and insulin resistance [11]. According to the International Diabetes Federation, approximately 11.1% of adults aged 20–79 – about 589 million people worldwide are living with diabetes (IDF Diabetes Atlas, 10th ed.). Lifestyle changes, diet and exercise programs can effectively manage T2DM when combined with pharmacotherapies such as insulin, metformin, sulfonylureas, thiazolidinediones and empagliflozin. In recent times, there has been an emergence of drugs such as sodium-glucose cotransporter 2 (SGLT2) inhibitors and glucagon-like peptide-1 receptor agonists (GLP-1 RAs) to promote glycemic control [12].

GLP-1 receptor agonists, including dulaglutide, liraglutide and semaglutide, are medications that mimic the action of the hormone GLP-1. These medications provide several benefits to the body: they boost insulin release when blood sugar is high, lower glucagon production, slow down gastric emptying and help reduce hunger by targeting the GLP-1 receptors in the brain [13–15]. Research has found that using these medications can lower the risk of experiencing a nonfatal stroke by 15%–16%. They also decrease the likelihood of serious heart issues, such as heart attacks and heart failure, and reduce the need for heart procedures like bypass surgery or stent placement. These positive effects are due to their ability to reduce inflammation, protect nerve cells, aid in weight loss and safeguard the blood vessels [16–18]. Because of these benefits, doctors are recommending GLP-1 receptor agonists for people with type 2 diabetes who are at a high risk of strokes and heart-related deaths.

Additionally, the effectiveness of GLP-1 medications is linked to dosage. In Asian individuals with type 2 diabetes, using these medicines for more than 251 days is connected to a lower risk of hospitalisation due to ischaemic stroke [19]. There is no one-size-fits-all dose for preventing strokes and heart issues, so treatment should be tailored to each person. It typically starts with a low dose, which is adjusted based on how well the individual responds to and tolerates the medication. These guidelines are supported by extensive research and align with the American Diabetes Association’s standards for diabetes treatment [16].

The motivation for incorporating GLP-1 receptor agonists into treatment plans for strokes in people with type 2 diabetes arises from the complex pathology of strokes in this population. While traditional treatments like blood thinners, anticoagulants and blood pressure medications are essential, they do not fully address the underlying blood vessel and inflammation issues in the brain that increase stroke risk in people with type 2 diabetes. This narrative review explores the integration of GLP-1 receptor agonists into treatment approaches, highlighting their added protective benefits against these associated risks.

## Methods

### Literature search strategy

Although this is a narrative review, a structured literature search was performed to comprehensively cover the relevant evidence. A literature search of PubMed/MEDLINE, Scopus and Web of Science was conducted for articles published in English until August 2025. The key words used were combinations of the following: ‘GLP-1 agonists’, ‘pathophysiology’, ‘stroke’, ‘stroke prevention’, ‘outcomes’, ‘complications’ and ‘therapy’. Further relevant articles were also manually sourced from the reference lists of related reviews and original studies. Human studies were prioritised, and those that directly related to the epidemiology, clinical presentation, diagnostic approach, management strategies or outcomes pertinent to GLP-1 agonists were favoured. Again, though not a systematic review per se, such measures were employed to ensure that, as far as possible, the narrative synthesis was transparent, rigorous and reproducible.

### Pathophysiology

A stroke occurs when an interruption of cerebral blood flow, whether from vascular occlusion or haemorrhage, leads to inadequate supply of oxygen and glucose to brain tissue. This triggers cellular energy failure, ionic pump dysfunction and ultimately neuronal death if circulation is not restored promptly [20]. In individuals with diabetes, this process is more intense because chronic hyperglycaemia accelerates endothelial dysfunction, promotes atherosclerosis and increases blood viscosity. These changes impair autoregulation of cerebral vessels and make arteries more susceptible to occlusion. Diabetes also enhances inflammatory and oxidative stress pathways, worsening ischaemic injury once a stroke begins. As a result, diabetic patients experience more severe strokes, poorer collateral circulation and slower recovery, highlighting the importance of early detection and aggressive risk-factor control [21].

In ischaemic stroke, obstruction of cerebral blood flow results in reduced oxygen and nutritional supply to a specific part of the brain. This leads to the development of a central ‘core’ and a surrounding ‘penumbra’. The core consists of dead neural tissue, while the penumbra has a metabolically compromised yet salvageable area that serves as the primary target for neuroprotective interventions [20]. The decrease in oxygen induces anaerobic glycolysis, which converts pyruvate into lactate, leading to acidosis. The reduction of blood flow to the cells reduces ATP levels, which impair Na and Ca ion pumps, especially in the core region [22]. Glutamate release increases the intracellular calcium (Ca^2+^) and sodium (Na^+^) levels inside cells, further lowering the ATP levels. Increased calcium inside the cell activates enzymes such as caspases, calpains and nitric oxide synthase. These enzymes produce damaging chemicals known as reactive oxygen species (ROS), which induce cell apoptosis [23]. The mitochondrial permeability transition pore (MPTP) is activated by elevated ROS and Ca^+^ levels, which result in the release of cytochrome c and the start of cell death pathways. Therefore, one of the main causes of brain injury is mitochondrial malfunction. After ischaemia, distress signals are released by core and penumbra cells, which cause inflammation [24]. Reperfusion makes inflammation worse. Hypoxia also triggers the clotting cascade, which includes complement system, endothelial cells and platelets. Pro-inflammatory chemicals such as cytokines (IL-1, IL-6, TNF), adhesion molecules (ICAM-1, VCAM-1), matrix metalloproteinases (MMPs) and prostaglandins are quickly activated [25]. Vasodilation is hampered, and thrombosis is encouraged by decreased nitric oxide production. Inflammation and oxidative stress weaken the blood-brain barrier (BBB), allowing immune cells to infiltrate and aggravate the injury.

Medications that target multiple pathways are essential because of the complex events, such as excitotoxicity, inflammation, and oxidative stress, that occur after ischaemia. In this setting, GLP-1 and its receptor agonists stand out as promising neuroprotectants. The GLP-1 receptor (GLP-1R) is widely expressed in various systems, including adipose tissue, the cardiovascular system and the gastrointestinal tract [26]. They are also distributed throughout the brain, found in the hippocampus, cortex, hypothalamus and brainstem. GLP-1, a 36-amino acid incretin hormone, is primarily produced by entero-endocrine L-cells in the distal small intestine and colon peripherally. In the central nervous system (CNS), preproglucagon neurons in the brainstem’s nucleus tractus solitarius (NTS) produce GLP-1 centrally and transport it to higher brain regions. This is crucial for preventing cerebral ischaemia and stroke, given its anti-inflammatory, antioxidant and anti-apoptotic effects [27,28]. GLP-1 and its agonists play a vital role in glucose metabolism by stimulating the release of insulin from pancreatic beta cells and inhibiting the release of glucagon from alpha cells in a glucose-dependent manner, thereby exerting their therapeutic effect on both diabetic and non-diabetic patients [29,30]. GLP-1RAs improve endothelial function and enhance vascular repair by increasing both the number and activity of circulating endothelial progenitor cells (EPCs). EPCs contribute to endothelial regeneration, angiogenesis and maintenance of vascular integrity by proliferating, migrating and integrating into damaged vessels, largely *via* the PI3K/Akt/eNOS signalling pathway. These effects strengthen microvascular function, support cerebral perfusion and provide a biologically plausible mechanism by which GLP-1RAs reduce ischaemic injury [31]

At the molecular level, GLP-1R activation in neuronal cells and glial cells increases intracellular cAMP. This increase in cAMP initiates protein kinase A (PKA) signalling, which activates the phosphoinositide 3-kinase (PI3K)/Akt pathway. This pathway helps assist in cell survival and inhibits apoptosis. It also inhibits nuclear factor-kappa B (NF-κB), a key mediator of inflammation. Moreover, GLP-1 preserves mitochondrial integrity by inhibiting MPTP-induced opening and cytochrome c release and strengthens antioxidant defences by synthesising enzymes like catalase and superoxide dismutase [32]. Central GLP-1 pathways support neurogenesis and synaptic plasticity, which are essential for penumbral recovery [33].

In insulin-resistant Apoe−/− Irs2+/− mice, GLP-1 receptor agonists, such as lixisenatide and liraglutide, have been found to enhance blood pressure control and glucose metabolism. Lixisenatide improved plaque stability by contributing to thicker fibrous caps, smaller necrotic cores and fewer inflammatory infiltrates; both treatments decreased the size of atheroma plaques. A shift towards anti-inflammatory M2 macrophages was linked to these benefits, suggesting that GLP-1 analogs lower cardiovascular and neurovascular risk *via* improving metabolic profiles and modifying immunological responses [34]. It has been shown that liraglutide reduces atherosclerosis *via* interacting with the macrophage scavenger receptor CD36, which promotes the uptake of oxidised low-density lipoprotein (LDL). Oxidised LDL in overabundance contributes to the development of foam cells and the advancement of atherosclerotic plaques. By stabilising plaques, liraglutide reduces the absorption of oxidised low-density lipoprotein (LDL), the production of foam cells and the risk of ischaemic stroke [35].

**Figure 1.**
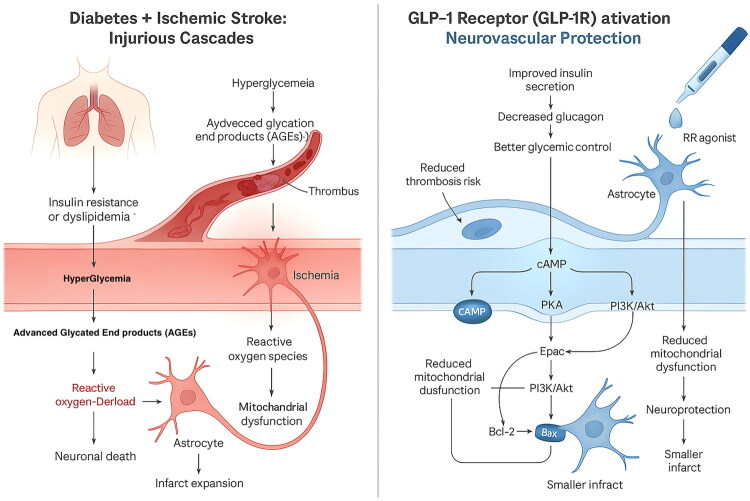
Pathophysiological mechanisms linking diabetes and ischaemic stroke and their modulation by GLP-1 receptor agonists. The pathogenesis driven by diabetes during stroke is described on the left. High blood sugar feeds AGEs formation, triggers oxidative stress, disrupts mitochondrial function, activates astrocytes and damages the endothelium, promoting clotting and pushing neurons towards apoptosis, leading to larger infarcts. On the right, GLP-1 receptor agonists depict their neurovascular protective effects: activation of the GLP-1 receptor enhances blood sugar control and diminishes thrombosis *via* turning on intracellular pathways including cAMP/PKA, Epac and PI3K/Akt. Thus, this leads to lower oxidative damage, healthier mitochondria, fewer pro-apoptotic signals with increased anti-apoptotic activity, all of which favour neuron survival and overall neuroprotection. Collectively, these actions decrease the area of neuronal damage following stroke.

GLP-1RAs reduce the pro-inflammatory aspects of cerebral atherosclerosis by lowering oxidative stress, apoptosis and the production and build-up of advanced glycation end products at the molecular and cellular levels. Additionally, GLP-1RA treatment directly improves endothelial function and stimulates angiogenesis, cerebral blood flow and neurogenesis. By indirectly lowering blood pressure, promoting weight loss and enhancing HbA1c control, GLP-1RAs minimise the risk of stroke [16]. GLP-1 receptor agonists may reduce the incidence of thrombotic events such as stroke by stabilising atherosclerotic plaques, improving endothelial function and reducing inflammatory macrophage activity. In conclusion, GLP-1 signalling promotes both stroke prevention and post-stroke neuronal protection through mechanisms that contribute to systemic metabolic and cardiovascular benefits. Figure 1 provides an overview of the mechanisms through which GLP-1 agonists help prevent stroke.

## Discussion

In this review, we synthesised evidence from cardiovascular outcome trials, meta-analyses, and mechanistic studies to evaluate the association between GLP-1 receptor agonist (GLP-1RA) therapy and cerebrovascular outcomes, with a particular focus on stroke risk reduction. Overall, the available clinical evidence suggests that GLP-1RAs may confer modest protection against nonfatal ischaemic stroke in patients with type 2 diabetes, although effect estimates are not uniform across individual trials and frequently reflect limited power for stroke-specific endpoints. Importantly, between-trial variability in patient risk profiles (primary vs secondary prevention, baseline cerebrovascular disease, and acute vs stable cardiovascular presentations), follow-up duration, treatment exposure and endpoint definition likely contribute to the observed heterogeneity in cerebrovascular findings. Accordingly, the interpretation of GLP-1RA–associated stroke effects should be grounded in a comparative appraisal of trial design and population characteristics rather than isolated point estimates from single studies.

Evidence from the Safe Implementation of Treatments in Stroke International Stroke Thrombolysis Register, which examined outcomes in over 16,000 patients receiving thrombolytic therapy, suggests that having diabetes increases the adjusted odds of mortality in stroke cases (odds ratio: 1.24; 95% CI: 1.07–1.44). Additionally, the odds of functional independence at 3 months after stroke were lower among patients with diabetes than non-diabetics (OR: 0.58; 95% CI: 0.48–0.70) [36].

Cardiovascular outcome trials (CVOTs) have consistently validated the safety of GLP-1RAs in individuals with type 2 diabetes, with several studies also demonstrating their potential to reduce stroke risk [37]. The beneficial effects of GLP-1RAs are best understood *via* their ability to mitigate pathophysiological mechanisms of diabetes, such as systemic inflammation, oxidative stress, endothelial dysfunction and vascular fibrosis, which are key contributors to cerebrovascular disease [38]. In this context, GLP-1RAs represent a promising addition to multifactorial risk management strategies, which are known to reduce the incidence of stroke. Their benefits include improved vascular risk markers, β-cell preservation [39,40], robust safety, high adherence and a low incidence of severe hypoglycaemia or significant adverse events. Across the CVOT trials for GLP-1 RAs, stroke has generally been assessed as a component of 3-point major adverse cardiovascular events (MACE) and/or as a secondary endpoint, with low absolute event counts in several trials and consequently wide confidence intervals. In long-acting GLP-1RA trials, point estimates for stroke were directionally favourable but variably precise. The LEADER trial (liraglutide), total stroke was not significantly reduced (HR 0.86, 95% CI 0.71–1.06), and nonfatal stroke similarly showed no significant between-group difference (HR 0.89, 95% CI 0.72–1.11), despite a significant reduction in the primary 3-point MACE composite (HR 0.87, 95% CI 0.78–0.97) [41]. Conversely, the SUSTAIN-6 trial reported a 39% reduction in the risk of nonfatal stroke with semaglutide (HR 0.61, 95% CI 0.38–0.99, *p* = 0.04), alongside benefits across cardiometabolic endpoints [42]. The Harmony Outcomes trial (albiglutide) did not demonstrate a significant effect on fatal or nonfatal stroke (HR 0.86, 95% CI 0.66–1.14) despite superiority for the MACE composite (HR 0.78, 95% CI 0.68–0.90). These findings in both LEADER and SUSTAIN-6 underscore the therapeutic potential across different classes of GLP-1RAs [43]. The REWIND trial further corroborated the evidence: dulaglutide decreased nonfatal stroke incidents by 24% in a broad patient population (HR 0.76; 95% CI, 0.61–0.95; *p* = 0.017), maintaining a favourable overall profile despite gastrointestinal side effects [44]. Similarly, the PIONEER trial, which evaluated oral semaglutide primarily for cardiovascular safety, demonstrated a non-significant yet favourable trend towards reduced nonfatal stroke incidence (HR 0.77; 95% CI, 0.56–1.05), consistent with findings from other GLP-1 receptor agonist studies [45]. In contrast, the ELIXA trial found no reduction in stroke incidence with lixisenatide in patients with acute coronary syndrome, highlighting variability in cerebrovascular outcomes across the class of GLP-1Ras [46]. Prespecified patient subgroups, endpoints and key study limitations of the included trials are summarised in Table 1.

**Table 1.** Comparison of GLP-1 receptor agonist trials: pre-specified patient subgroups, endpoints and key study limitations.

Trial	Patient population and pre-specified subgroups	Primary endpoint	Key cardiovascular results (summary)	Major study limitations
LEADER (liraglutide, s.c. daily)	Adults with type 2 diabetes and high cardiovascular risk. Pre-specified subgroup analyses by age, sex, region, race/ethnicity, BMI, baseline HbA1c, diabetes duration, baseline cardiovascular disease, heart failure, prior myocardial infarction or stroke, kidney function (including eGFR strata) and background glucose-lowering therapy.	3-point MACE: cardiovascular death, nonfatal myocardial infarction or nonfatal stroke.	Liraglutide reduced risk of MACE versus placebo (HR ≈0.87). The effect was mainly driven by lower rates of cardiovascular death and all-cause mortality, with more modest, nonsignificant reductions in nonfatal myocardial infarction and nonfatal stroke. Microvascular benefits were observed, largely driven by reduced nephropathy events.	Enriched high-risk population limits generalizability to lower-risk type 2 diabetes. Many secondary and exploratory outcomes (including microvascular endpoints) were not adjusted for multiplicity. Changes in background therapies over long follow-up may have attenuated or modified treatment effects. Subgroup findings (e.g. greater benefit at lower eGFR) are hypothesis-generating rather than definitive.
SUSTAIN-6 (semaglutide, s.c. weekly)	Adults with type 2 diabetes and established cardiovascular disease, chronic kidney disease or multiple cardiovascular risk factors. Randomization stratified by cardiovascular disease status, insulin use and kidney function. Subgroups evaluated by these and other baseline characteristics.	3-point MACE (cardiovascular death, nonfatal myocardial infarction or nonfatal stroke), designed primarily as a cardiovascular safety (noninferiority) trial.	Semaglutide reduced MACE versus placebo (HR ≈0.74), with a particularly notable reduction in nonfatal stroke. Cardiovascular and all-cause mortality were numerically but not significantly lower. An increased risk of diabetic retinopathy complications was observed, particularly among patients with pre-existing retinopathy and rapid glycemic improvement.	Event-driven, relatively short follow-up (∼2 years), and design focused on noninferiority rather than definitive superiority. Limited power for detailed subgroup analyses. The retinopathy signal requires cautious interpretation due to potential confounding by baseline eye disease and rapid HbA1c reduction. Multiple secondary outcomes were not fully controlled for multiplicity.
REWIND (dulaglutide, s.c. weekly)	Broad type 2 diabetes population with and without established cardiovascular disease (majority without prior events). Prespecified subgroup analyses by age, sex, BMI, diabetes duration, baseline HbA1c, prior cardiovascular disease and geographic region.	3-point MACE (cardiovascular death, nonfatal myocardial infarction or nonfatal stroke).	Dulaglutide significantly reduced MACE (HR ≈0.88). The benefit was consistent in patients with and without established cardiovascular disease. The strongest component signal was a reduction in nonfatal stroke, with more modest effects on myocardial infarction and cardiovascular death.	Long follow-up with substantial treatment discontinuation and intensification of background therapies in both arms, which may dilute treatment effects. Multiple subgroup comparisons elevate risk of chance findings; interaction results should be viewed as supportive rather than confirmatory.
HARMONY OUTCOMES (albiglutide, s.c. weekly)	Adults with type 2 diabetes and established cardiovascular disease. Prespecified subgroups included age, sex, race/ethnicity, geographic region, diabetes duration, BMI, baseline HbA1c, kidney function, prior coronary/cerebrovascular/peripheral artery disease, heart failure history and background cardiovascular and glucose-lowering treatments.	3-point MACE (cardiovascular death, myocardial infarction or stroke).	Albiglutide significantly reduced MACE versus placebo (HR ≈0.78), with broadly consistent effects across most examined subgroups. The absolute risk reduction was substantial, reflecting high baseline event rates.	Median follow-up was relatively short (about 1.6 years) compared with other GLP-1 trials, limiting assessment of long-term efficacy and safety. Subgroup interaction tests were not adjusted for multiple comparisons, increasing the risk of spurious subgroup effects. The drug was subsequently withdrawn from the market for commercial, not safety, reasons, which may affect clinical uptake.
EXSCEL (exenatide, once-weekly)	Adults with type 2 diabetes, with or without prior cardiovascular disease (approximately three-quarters had established cardiovascular disease). Subgroups prespecified by age, sex, race, region, diabetes duration, baseline HbA1c, BMI, kidney function, prior cardiovascular event and heart failure history.	3-point MACE (cardiovascular death, nonfatal myocardial infarction or nonfatal stroke) with hierarchical testing for noninferiority then superiority.	Exenatide once weekly was noninferior to placebo for MACE (HR ≈0.91) but did not achieve statistical superiority. All-cause mortality was modestly reduced, whereas individual MACE components showed neutral to borderline favourable trends.	High rates of treatment discontinuation and variable exposure may bias findings toward the null and complicate interpretation of efficacy. The primary confidence interval was close to 1.00, providing strong reassurance on safety but limited evidence for robust benefit. The hierarchical testing scheme restricts formal inference on secondary endpoints once superiority for MACE is not met.
PIONEER-6 (oral semaglutide)	Adults with type 2 diabetes at high cardiovascular risk. Designed primarily as a pre-approval cardiovascular safety trial with a modest number of expected events and short follow-up. Subgroup analyses were exploratory.	3-point MACE (cardiovascular death, nonfatal myocardial infarction or nonfatal stroke), with a noninferiority framework.	Oral semaglutide met noninferiority for MACE (HR ≈0.79). Numerically fewer cardiovascular and all-cause deaths were observed in the semaglutide group, but the confidence intervals were wide, and the trial was not powered for definitive superiority. Stroke and myocardial infarction components were directionally favourable but not statistically significant.	Relatively small number of MACE events and short follow-up limit precision and power, especially for component endpoints and subgroup analyses. The trial was designed to demonstrate safety rather than conclusive cardiovascular benefit; secondary endpoint and subgroup findings are exploratory and not adjusted for multiplicity.
ELIXA (lixisenatide, s.c. daily; post-ACS)	Adults with type 2 diabetes and a recent acute coronary syndrome. Subgroup analyses included age, sex, kidney function, geographic region and cardiovascular history (including heart failure), but no major treatment interactions were detected.	Composite of cardiovascular death, nonfatal myocardial infarction, nonfatal stroke or hospitalization for unstable angina.	Lixisenatide was neutral on the primary composite endpoint (HR ≈1.02), meeting noninferiority but showing no evidence of superiority. Individual components, including stroke, myocardial infarction and cardiovascular death, were all neutral.	Restriction to a post-ACS population limits generalizability to stable, chronic cardiovascular disease. Follow-up of about 2 years may be insufficient to detect long-term vascular or renal effects. The neutral result, combined with multiple subgroup and secondary analyses, means that any apparent subgroup differences are likely chance findings.
FIGHT (liraglutide in HFrEF)	Patients with heart failure with reduced ejection fraction (HFrEF), with and without diabetes. Subgroup analyses by diabetes status did not demonstrate significant interaction.	Global rank primary endpoint incorporating death, heart failure hospitalization, and changes in biomarkers and functional status at 180 days (HF-focused composite rather than classic atherosclerotic MACE).	Liraglutide did not improve the primary global rank endpoint versus placebo. Secondary outcomes and exploratory analyses showed no convincing evidence of benefit and suggested a possible increase in heart-failure–related events in some analyses, particularly among patients with diabetes, although confidence intervals were wide.	Modest sample size and limited follow-up (approximately 6 months) restrict power to detect differences in clinical events. The complex global rank endpoint can be difficult to interpret clinically. The trial was not powered for detailed safety or subgroup analyses; any apparent excess of HF events should be considered hypothesis-generating.

Long-acting versus short-acting GLP-1RAs differ not only in dosing interval but also in the pattern of receptor stimulation and the vascular risk factors they preferentially modify. Short-acting agents (e.g. lixisenatide) provide intermittent exposure with a predominantly prandial glycemic profile, with comparatively greater post-prandial glucose effects driven by sustained gastric emptying delay. In contrast, long-acting agents (liraglutide, semaglutide, dulaglutide, albiglutide and long-acting exenatide formulations) provide more continuous receptor engagement and generally yield more durable improvements in fasting glycaemia, body weight and blood pressure, intermediate traits that correlate with ischaemic stroke risk [47]. This pharmacologic distinction provides a biologically plausible framework for why several long-acting GLP-1RAs demonstrate directionally favourable stroke estimates, whereas short-acting lixisenatide in a post-ACS population did not reduce stroke incidence in ELIXA [46]. However, duration of action alone is insufficient to explain cross-trial variability, because long-acting agents show heterogeneous cerebrovascular findings (e.g. significant nonfatal stroke reduction in SUSTAIN-6 and REWIND, but neutral stroke components in LEADER and HARMONY), indicating that molecule-specific factors and trial architecture also materially influence detection of stroke effects [41–44].

In contrast, in the FIGHT trial, liraglutide did not show any improvement in clinical stability, outcomes or biomarkers in patients with advanced heart failure and a reduced ejection fraction, suggesting no cerebrovascular or cardioprotective benefits in this group. These results emphasise the necessity for personalised treatment based on comorbidities and clinical profile [48]. Similarly, the EXSCEL trial revealed that once-weekly exenatide did not significantly lower stroke incidence in patients with type 2 diabetes, though a favourable trend was observed. Although cardiovascular safety was established with GLP-1 receptor agonists, the findings suggest that stroke-related benefits may vary across different GLP-1 receptor agonists. This highlights the need to individualise therapy based on patient-specific risk factors and treatment goals, especially when stroke prevention is a key concern [49].

Discordant cerebrovascular outcomes across GLP-1RA CVOTs are likely multifactorial. First, stroke was typically a secondary or component endpoint with low absolute event counts in several trials, limiting power and yielding wide confidence intervals; this is particularly relevant for shorter follow-up trials (e.g. HARMONY and PIONEER), where modest true effects may not be detectable or appear inconsistent across studies [43,45]. Second, enrolled populations differed substantially with respect to baseline cerebrovascular risk and clinical context (recent ACS in ELIXA vs chronic stable atherosclerotic disease vs mixed primary prevention in REWIND), which can alter stroke subtype distribution and the time course over which vascular risk modification translates into event reduction [44,46]. Third, endpoint definitions varied (fatal/nonfatal vs nonfatal-only stroke, and differing adjudication frameworks within composite endpoints), limiting direct comparability across trials. Fourth, treatment separation and exposure differed, with discontinuation and background cardioprotective therapy potentially diluting incremental stroke effects in intention-to-treat analyses, especially over longer follow-up periods.

### Evidence synthesis and research gaps

While substantial evidence supports the cardiovascular safety of GLP-1 receptor agonists (GLP-1RAs), interpreting their specific benefits regarding stroke requires caution due to the varying outcomes across clinical trials. Recent meta-analyses have sought to address this heterogeneity and clarify the role of GLP-1RAs in reducing cerebrovascular risk among individuals with type 2 diabetes (T2D). A comprehensive meta-analysis, which included 28 randomised controlled trials with over 74,000 participants, found that GLP-1RA use was linked to a statistically significant reduction in adverse cerebrovascular events (RR 0.83; 95% CI, 0.76–0.91), particularly for nonfatal and ischaemic strokes, while no benefit was observed for haemorrhagic or fatal strokes. Subgroup analyses revealed that longer-acting, human GLP-1-based agents, such as dulaglutide and semaglutide, provided more consistent benefits, whereas shorter-acting exendin-4-based agents, like lixisenatide, showed neutral outcomes. Notably, the cerebrovascular protective effects were more pronounced in individuals with preserved renal function and shorter duration of diabetes, suggesting potential modifiers of treatment response [50]. A separate meta-analysis of 11 CVOTs involving over 82,000 participants reported a 16% relative reduction in total stroke risk with GLP-1RA therapy compared to placebo, with consistent decreases in nonfatal strokes (RR 0.87; 95% CI, 0.79–0.95), but no significant effect on fatal stroke risk [18]. Importantly, these benefits were independent of the route (oral vs. subcutaneous), frequency (daily vs. weekly) or presence of diabetes, thereby supporting a broader indication for stroke prevention, beyond glucose control alone. Additionally, a recent meta-analysis of 13 trials investigating both GLP-1RAs and dual GIP/GLP-1 receptor agonists such as tirzepatide found similar reductions in major adverse cardiovascular events (MACE) and stroke, particularly ischaemic stroke (OR 0.74; 95% CI, 0.61–0.91). Again, GLP-1RAs showed no effect on haemorrhagic stroke (OR 0.92; 95% CI, 0.51–1.66), reinforcing their cerebrovascular benefit profile as predominantly ischaemic-specific [51].

Together, these analyses provide compelling evidence that GLP-1RAs, especially longer-acting human GLP-1 analogs, are effective in reducing the risk of ischaemic stroke in patients with T2DM, with a favourable safety profile. However, the modest absolute risk reductions highlight the importance of carefully selecting patients and stratifying risks before initiating these drugs. Factors such as eGFR, diabetes duration and history of stroke subtype may help personalise therapy. In addition, the population-level impact of GLP-1RAs will be influenced by implementation factors, including drug acquisition cost, insurance coverage/prior-authorization requirements and supply constraints that can limit initiation and long-term persistence, particularly in socioeconomically disadvantaged groups. Consequently, cost-effectiveness analyses and real-world effectiveness studies that incorporate adherence and discontinuation are needed to contextualise cerebrovascular benefit under routine-care conditions. Table 2 summarises all the available GLP-1s in the market along with their pharmacokinetics, indications and safety profile.

**Table 2.** Comparison of GLP-1 receptor agonists: pharmacokinetics, dosing, approved indications and cardio-cerebrovascular outcomes.

GLP-1 RA	Pharmacokinetics	Dosing frequency	FDA-approved indications	Cardiovascular outcomes	Cerebrovascular outcomes	Key safety considerations
Liraglutide	Long-acting; t½ ∼13h; continuous receptor activation	Once daily SC	T2DM glycemic control CV risk reduction in T2DM with established CVD Chronic weight management	MACE reduction 13% (HR 0.87); CV death reduction; all-cause mortality reduction	Numerically lower stroke rates	GI adverse events common; weight loss 2–4 kg
Semaglutide (SC)	Long-acting; t½ ∼1 week; weekly dosing	Once weekly SC	T2DM glycemic control CV risk reduction in T2DM with established CVD	MACE reduction 26% (HR 0.74); nonfatal stroke reduction	Reduced nonfatal stroke; ischemic stroke reduction	GI adverse events; retinopathy concerns in some trials
Semaglutide (oral)	Long-acting; similar PK to SC formulation	Once daily oral	T2DM glycemic control	CV death reduction; favourable MACE trend (HR 0.79, non-inferiority met)	Reduced ischemic stroke/TIA composite	First oral GLP-1 RA; GI adverse events
Dulaglutide	Long-acting; t½ ∼5 days	Once weekly SC	T2DM glycemic control (adults and pediatric ≥10 years) CV risk reduction in T2DM with established CVD or multiple risk factors	MACE reduction 12% (HR 0.88); benefits in primary prevention population (68.5% without established CVD)	Reduced stroke (HR 0.76); ischemic cerebrovascular event reduction	GI adverse events; studied in broader primary prevention population
Exenatide	Short-acting (twice daily) or long-acting (weekly); variable t½	Twice daily or once weekly SC	T2DM glycemic control	No significant MACE reduction in EXSCEL (HR 0.91, NS); numerically lower CV events	No significant stroke reduction	Greater postprandial glucose effect (short-acting); GI adverse events
Lixisenatide	Short-acting; t½ 2-3h; based on exendin-4	Once daily SC	T2DM glycemic control	No CV benefit in ELIXA (HR 1.02); neutral on MACE	Greater stroke events vs. other GLP-1 RAs; no ischemic stroke benefit	Primarily postprandial glucose lowering; pronounced gastric emptying delay
Albiglutide	Long-acting; weekly dosing	Once weekly SC	T2DM glycemic control (discontinued from market)	MACE reduction in HARMONY (HR 0.78); fewer MI events vs. lixisenatide	Fewer retinopathy events vs. semaglutide	No longer commercially available
Tirzepatide	Dual GIP/GLP-1 agonist; long-acting	Once weekly SC	Obesity/weight management Moderate-severe OSA in obesity	Under investigation for CV outcomes; HF benefits in SUMMIT trial	Limited cerebrovascular data available	Superior weight loss vs. traditional GLP-1 RAs; GI adverse events

## Limitations

Despite encouraging signals for ischaemic stroke risk reduction, several evidence gaps limit inference and generalisability. First, most cardiovascular outcome trials were not designed with stroke as a primary endpoint; stroke was typically a secondary or component outcome with limited event counts and stroke subtypes and mechanisms were inconsistently collected or reported, constraining mechanistic interpretation and subgroup identification. Second, the evidence base is vulnerable to selective reporting and publication bias, and the exclusion of non-English and unpublished/grey literature may preferentially omit null findings; future syntheses should expand searches to trial registries and regulatory sources and assess small-study effects when feasible. Third, while GLP-1RAs are well studied in type 2 diabetes, data for primary prevention in non-diabetic but high-risk individuals remain sparse, including patients with prior ischaemic stroke or transient ischaemic attack and those with high vascular risk driven by obesity, metabolic syndrome, or chronic kidney disease. As a result, the extent to which observed cerebrovascular benefits represent glycaemia-independent effects versus risk-factor modification in diabetes cannot be fully disentangled. Fourth, real-world effectiveness data are limited, and observational studies are needed to evaluate generalisability, treatment persistence, and safety in routine practice while addressing confounding and time-varying therapy. Finally, the literature provides insufficient granularity for stratified inference across key effect modifiers. Specifically, robust assessments by ethnicity/race, socioeconomic status and social determinants of health are limited, despite known disparities in stroke incidence, access to preventive therapies and background risk-factor control. Similarly, comparative and combination-effect data remain underdeveloped, including head-to-head comparisons of GLP-1RAs against other cerebroprotective strategies and evaluations of concomitant medications (notably SGLT2 inhibitors, statins, antihypertensives and antithrombotics) that may modify baseline risk and treatment effect. A trial evaluating the broader physiological impacts of semaglutide, including effects on cardiovascular function, renal function and insulin sensitivity in adults, is currently active [52]. New oral small-molecule agents (e.g. orforglipron) are in advanced phase 3 development for diabetes and weight loss, with broader cardiovascular outcomes including stroke risk expected to be evaluated as part of larger registrational programs [53]. Future studies should focus on properly designed trials that specifically address the impact on stroke prevention, optimise predictive markers of response and consider application to non-diabetic populations with stroke.

## Conclusion

GLP-1RAs represent a significant advancement in the prevention and management of ischaemic stroke among individuals with T2DM. Clinical evidence, including that derived from cardiovascular outcome trials and large meta-analyses, clearly demonstrates that these agents significantly lower the incidence of nonfatal ischaemic stroke. Their therapeutic efficacy mainly arises from the diverse biological effects of these agents, including anti-inflammatory, antioxidant, neuroprotective and endothelial-protective properties. Notably, long-acting, human GLP-1 analogs (e.g. semaglutide and dulaglutide) have consistently superior stroke prevention benefits vs short-acting variants. Nevertheless, variability in outcomes between individual studies highlights the need for careful patient selection and personalised therapeutic approaches, particularly in light of factors such as renal function, diabetes duration and cerebrovascular risk profiles. Despite strong evidence for their cerebrovascular protective effects in diabetic populations, significant gaps still exist. Future studies should target stroke prevention as their primary endpoint. Here, detailed patient stratification, identification of predictive biomarkers for therapeutic responsiveness and an assessment of the potential extension of these medications beyond classical glycaemic control into wider frameworks of cardiometabolic risk management are warranted. Ultimately, integrating GLP-1RAs into comprehensive stroke prevention strategies may significantly enhance clinical outcomes and quality of life for patients at elevated cerebrovascular risk.

## Data Availability

Data sharing is not applicable to this article as no data was created or analysed in this research.
